# Input transformation by dendritic spines of pyramidal neurons

**DOI:** 10.3389/fnana.2014.00141

**Published:** 2014-12-02

**Authors:** Roberto Araya

**Affiliations:** Department of Neurosciences, Faculty of Medicine, University of MontrealMontreal, QC, Canada

**Keywords:** spine neck, synaptic transmission, plasticity, synaptic integration, biophysical processes, two-photon uncaging, dendritic computation, input-output transformation

## Abstract

In the mammalian brain, most inputs received by a neuron are formed on the dendritic tree. In the neocortex, the dendrites of pyramidal neurons are covered by thousands of tiny protrusions known as dendritic spines, which are the major recipient sites for excitatory synaptic information in the brain. Their peculiar morphology, with a small head connected to the dendritic shaft by a slender neck, has inspired decades of theoretical and more recently experimental work in an attempt to understand how excitatory synaptic inputs are processed, stored and integrated in pyramidal neurons. Advances in electrophysiological, optical and genetic tools are now enabling us to unravel the biophysical and molecular mechanisms controlling spine function in health and disease. Here I highlight relevant findings, challenges and hypotheses on spine function, with an emphasis on the electrical properties of spines and on how these affect the storage and integration of excitatory synaptic inputs in pyramidal neurons. In an attempt to make sense of the published data, I propose that the *raison d'etre* for dendritic spines lies in their ability to undergo activity-dependent structural and molecular changes that can modify synaptic strength, and hence alter the gain of the linearly integrated sub-threshold depolarizations in pyramidal neuron dendrites before the generation of a dendritic spike.

## Introduction

**Figure 1:**
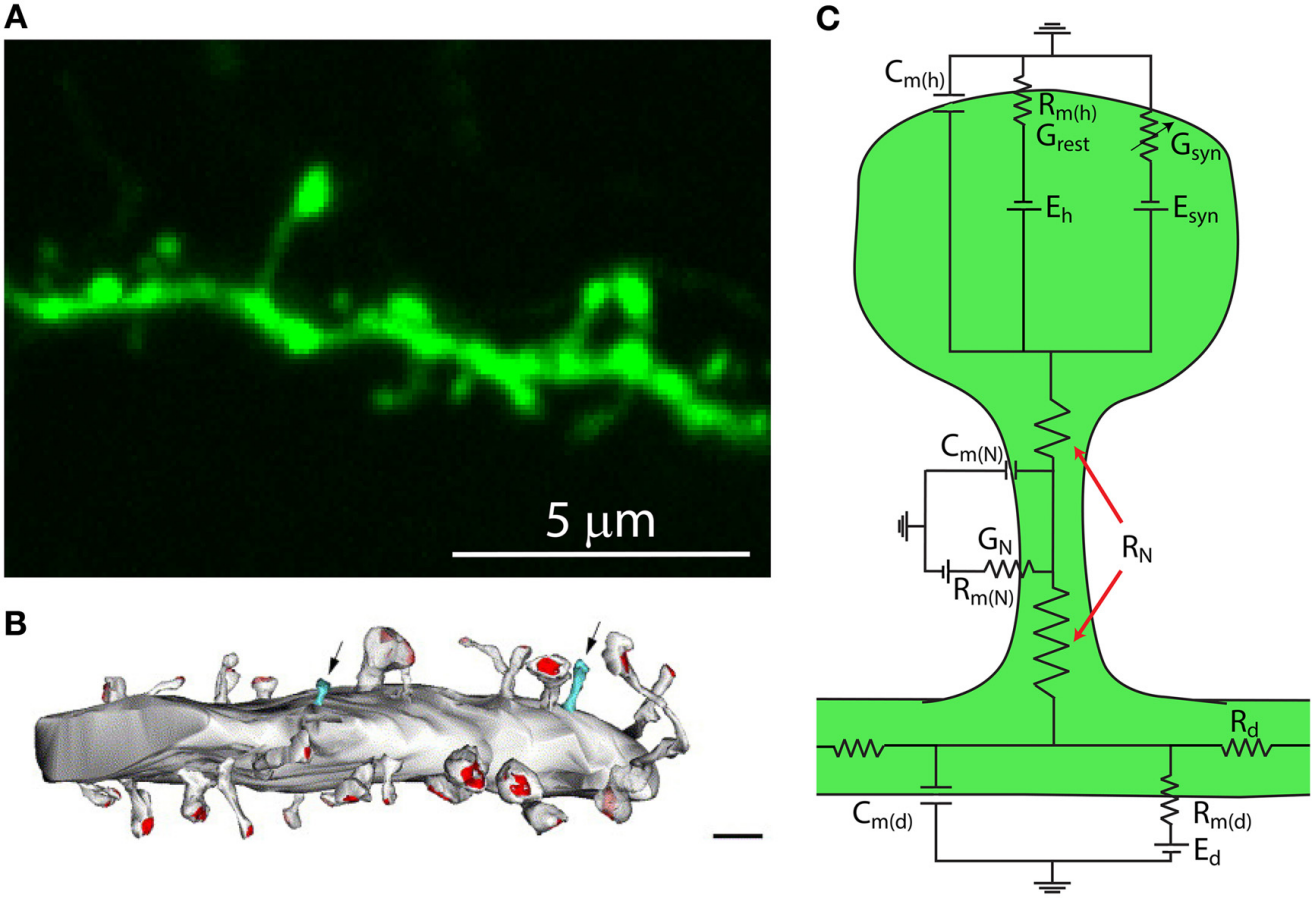
**Dendritic spines are tiny protrusions that cover the dendrites of pyramidal neurons and are the sites at which excitatory connections are made. (A)** Confocal scanning image of a representative dendrite, covered with dendritic spines, of a layer 5 pyramidal neuron from a thy1-YFP-H transgenic mouse expressing the yellow fluorescent protein. **(B)** Synaptic contacts occur at spines. Reconstruction of electron micrographs taken from serial sections of dendritic segments from neocortical pyramidal neurons. Note the distribution of postsynaptic contacts (PSD, red; excitatory asymmetric contacts). Only a few percent of dendritic protrusions are devoid of synaptic contacts (blue). Note that the shaft lacks excitatory synaptic contacts. Scale bar = 2 μm (Modified with permission from Arellano et al., 2007b). **(C)** Simplified circuit diagram of a passive dendritic spine. *C*_*m*(*h*)_, capacitance of the spine head membrane; *C*_*m*(*N*)_, capacitance of the spine neck membrane; *C*_*m*(*d*)_, dendritic membrane capacitance; *R*_*m*(*h*)_, membrane resistance of the spine head; *R*_*m*(*N*)_, membrane resistance of the spine neck; *R*_*m*(*d*)_, membrane resistance of the dendrite; *E_h_*, reversal potential at the spine head; *E_syn_*, synaptic reversal potential; *E_d_*, reversal potential at the dendrite; *R_N_*, neck resistance; *R_d_* dendritic resistance; *G_rest_*, spine's conductance at rest; *G_syn_*, spine's synaptic conductance; *G_N_*, spine neck's conductance.

The fundamental operation of a neuron is to integrate synaptic inputs and decide whether and when to fire an action potential. Neocortical neurons, which may be subdivided into glutamatergic pyramidal neurons and GABAergic interneurons, form complex networks that are ultimately responsible for the production of higher cognitive functions (Kandel et al., 2000). The pyramidal neuron is the most abundant neuron in the cerebral cortex, yet how it processes, stores, and integrates its thousands of inputs remains ill-defined. A notable characteristic of pyramidal neurons is that their dendrites are covered by tiny protrusions called dendritic spines (Figures 1A,B). Since their discovery by Santiago Ramon y Cajal in 1888 (Cajal, 1888) we have learned a great deal about their morphological, molecular and biophysical properties. Dendritic spines are the main gateway of excitatory synaptic transmission in the brain (Gray, 1959), with almost all (~95%) of excitatory synaptic input to pyramidal neurons being received by spines (Spacek and Harris, 1998; Arellano et al., 2007b; Chen et al., 2012a) (Figure 1B). In addition, it has been shown that GABAergic inputs target not only dendritic shafts but also some dendritic spines (Somogyi and Cowey, 1981; Freund et al., 1986; DeFelipe et al., 1989; Chen et al., 2012a), with recent evidence indicating that GABAergic synapses from somatostatin-expressing interneurons can be directed to some spine heads, exerting a local inhibition of Ca^2+^ signals (Chiu et al., 2013).

Although excitatory input to GABAergic interneurons does not show the same exclusivity for spines, these structures are also present on various classes of interneurons (Feldman and Peters, 1978; Freund and Buzsaki, 1996; Kawaguchi et al., 2006) and share some of the functional properties of spines found on pyramidal neurons (Scheuss and Bonhoeffer, 2013). The issue of why the prevalence of dendritic spines varies between interneuron subtypes is intriguing but is not covered in this review; the reader is referred to the following references for further information (Feldman and Peters, 1978; Kawaguchi, 1993; Pitkanen and Amaral, 1993; McBain et al., 1994; Freund and Buzsaki, 1996; Kawaguchi et al., 2006; Keck et al., 2011; Scheuss and Bonhoeffer, 2013).

Importantly, spines are believed to be the preferential site for the induction of synaptic long-term potentiation (LTP) (Lang et al., 2004; Matsuzaki et al., 2004; Harvey et al., 2008; Araya et al., 2014) and can undergo structural remodeling. In addition, several studies have demonstrated that dendritic spines are the fundamental substrates of pathogenesis in neuropsychiatric disorders such as the autism spectrum disorders, schizophrenia, and neurodegenerative disorders such as Alzheimer's disease (Selemon and Goldman-Rakic, 1999; Glantz and Lewis, 2000; Tackenberg et al., 2009; Hutsler and Zhang, 2010; Portera-Cailliau, 2012), which are characterized by impairments in spine structure and/or density (Penzes et al., 2011).

Although the importance of spines is acknowledged, the biophysical and molecular mechanisms controlling their function in health and disease remain poorly understood. Their small size (<1 fL volume) has allowed us only indirect access for measurement using standard electrophysiological techniques. However, with the advent of two-photon (2P) microscopy (Denk et al., 1990), and the development of 2P glutamate uncaging—with which it is possible to image and photo-activate live dendritic spines deep in tissue with high spatial resolution (Matsuzaki et al., 2001; Araya et al., 2006b; Bloodgood and Sabatini, 2007; Harvey et al., 2008)—some experimental challenges posed by the small size of dendritic spines have been bypassed. In combination with virally delivered or genetically encoded fluorescent Ca^2+^ indicators (Nakai et al., 2001), voltage sensitive dyes (Peterka et al., 2011), fluorescence resonance energy transfer (FRET)-based sensors (Yasuda, 2006; Lee et al., 2009; Murakoshi et al., 2011), and optogenetic activation and inactivation techniques (Fenno et al., 2011), we have begun to gather important information as to how spine function relates to the input/output properties of excitatory neurons. Here I will review what we know about spines, with an emphasis on their electrical properties and how passive (e.g., spine morphology) and active mechanisms (recruitment of voltage-gated spine channels) might affect the storage and integration of excitatory inputs in pyramidal neurons.

## Structural properties of spines in pyramidal neurons: use of nanoscopy methods

The peculiar morphology of spines (Cajal, 1888), with their small head (~1 μm in diameter and <1 fL volume) separated from the main dendrite by a slender neck (<0.2 μm in diameter) (Sorra and Harris, 2000; Arellano et al., 2007b; Takasaki and Sabatini, 2014; Tonnesen et al., 2014) (Figure 1), has inspired decades of theoretical work that has, in the past 25 years, been complemented by much needed experimental work. Together, these efforts have been aimed at understanding how a synaptic current at the spine head, and the voltage signal generated by it, are delivered to the parent dendrite, as well as the effect that spine morphological properties have on the integration of excitatory inputs (Chang, 1952; Rall, 1964; Llinás and Hillman, 1969; Jack et al., 1975; Koch and Poggio, 1983a,b; Segev and Rall, 1988). In addition, the extent to which the morphological, molecular and biophysical properties of spines transform synaptic inputs (Miller et al., 1985; Araya et al., 2006b; Harnett et al., 2012), support synaptic plasticity (Matsuzaki et al., 2004; Harvey et al., 2008; Araya et al., 2014), and affect the integration of excitatory synaptic inputs (Llinás and Hillman, 1969; Araya et al., 2006a) have been intensively investigated.

Ultrastructural studies using electron microscopy (EM) or super-resolution light microscopy (see below) have shown that different spine shapes co-exist in the dendrites of pyramidal neurons (see Table 1). Spines can be classified morphologically as “stubby” (lacking a neck), “thin” (thin, long neck with an apparent head), or “mushroom” (big head with thick neck) (Peters and Kaiserman-Abramof, 1970), and activity-dependent changes in their morphology (Lang et al., 2004; Matsuzaki et al., 2004; Harvey et al., 2008; Tanaka et al., 2008; Araya et al., 2014) and/or internal biochemistry (Matsuzaki et al., 2004; Yasuda and Murakoshi, 2011; Sala and Segal, 2014) are thought to affect synaptic efficacy. In this way, spines may serve as substrates for synaptic plasticity and be a means through which the input/output properties of pyramidal neurons are altered (Lang et al., 2004; Matsuzaki et al., 2004; Harvey et al., 2008; Araya et al., 2014). In electron microscopy (EM), electrons are the source of illumination and hence the resolving power is much higher than with light microscopy; this has allowed us to learn a great deal about spine architecture and spine morphological variability at the nanoscale (Gray, 1959; Spacek and Harris, 1998; Sorra and Harris, 2000; Arellano et al., 2007a). However, the use of EM necessitates using fixed tissue, since the sample needs to be permeabilized, fixated, dehydrated, and placed under high vacuum. In addition, fixation and embedding protocols may cause structural artifacts (compare the morphological discrepancies of images from live spines with their respective images gathered using EM in Knott et al., 2006 and Chen et al., 2012a).

**Table 1:** **Dimensions of individual spines with electron microscopy and STED imaging**.

**CA1 pyramidal cell**	**EM (fixed tissue)**	**STED (live tissue)**
Neck length (μm)	–	0.157–1.8 (0.689)^*^
Neck width (μm)	0.038–0.46^b^	0.059–0.292 (0.167)^*^
Head width (μm)	–	0.262–1.104 (0.583)^*^
Head volume (μm^3^)	0.003–0.55^b^	–
**Cortical pyramidal cell**		
Neck length (μm)	0.1–2.21 (0.66)^a^	–
Neck width (μm)	0.09–0.51 (0.2)^a^	–
Head width (μm)	–	–
Head volume (μm^3^)	0.01–0.38 (0.07)^a^	–

* *Tonnesen et al. (2014)*.

a *Arellano et al. (2007a)*.

b *Sorra et al., (Sorra and Harris, 2000)*.

Recent developments in super-resolution light microscopy techniques have enabled us to perform sub diffraction-limited imaging of live dendritic spines (for review see Huang et al., 2010; Maglione and Sigrist, 2013). Among the main nanoscopy methods utilized to study brain structures are stimulated emission depletion (STED) microscopy, photoactivatable localization microscopy (PALM) (Betzig et al., 2006; Hess et al., 2006) and stochastic optical reconstruction microscopy (STORM) (Rust et al., 2006). In stimulated emission depletion (STED) microscopy the fluorescence emission at the border of the point-spread function (PSF) of the microscope is depleted by creating an annulus of high intensity light overlaying the outer edge of the PSF, through a process known as stimulated emission depletion. This leaves a fluorescence volume only at the center of the PSF, providing lateral and axial resolutions of 30–50 nm and ~30–600 nm, respectively (Huang et al., 2010; Maglione and Sigrist, 2013). In photoactivated localization microscopy (PALM) (Betzig et al., 2006; Hess et al., 2006) and stochastic optical reconstruction microscopy (STORM) (Rust et al., 2006), detection of images beyond the diffraction-limited resolution is achieved by using photoswitching or other mechanisms to stochastically activate individual fluorophores that are separated by a distance greater than the diffraction-limited resolution of the microscope, allowing their individual localization. Image reconstruction is obtained by superimposing a large number of wide-field images, each containing only a few individually detected fluorophores (Huang et al., 2010; Maglione and Sigrist, 2013). PALM uses fluorophores in the form of photoactivatable fluorescence proteins, while STORM uses immunolabeling with cyanine-tagged dyes (Huang et al., 2010; Maglione and Sigrist, 2013). Although some laboratories have successfully used PALM and STORM to image live brain tissue (Dani and Huang, 2010) and spines (Lu et al., 2014), their low imaging speed hinder the collection of high resolution images in live samples. However with STED, small fields of view can be imaged rapidly, and when combined with 2P-excitation optical sectioning one can image at considerable depths (~80–100 μm) in thick acute brain slices (Bethge et al., 2013; Takasaki et al., 2013). Thus, STED allows imaging of live dendritic spines, providing a super-resolution view of the spine neck (length and diameter) and head (see Table 1) (Nagerl et al., 2008; Nagerl and Bonhoeffer, 2010; Maglione and Sigrist, 2013; Takasaki and Sabatini, 2014; Tonnesen et al., 2014) and thus enabling an improved assessment of the spine structure–function relationship. Although the benefits of STED and PALM/STORM are evident, their current disadvantage is the need for high fluorescence labeling density in order to collect many photons per pixel to provide an acceptable signal-to-noise ratio (Maglione and Sigrist, 2013). In STED microscopy, the use of continuous wave lasers requires higher depletion beam power than with pulsed lasers, resulting in more severe photobleaching of the sample (Willig et al., 2007). Some of these constrains have been bypassed by the use of Switching Laser Mode (SLAM) microscopy, in which switching between laser modes in a confocal microscope provides a way for diffraction-limited resolution images of spines and other structures to be enhanced by a factor of two. To obtain images of sub-diffraction resolution and contrast, it is necessary to subtract the images obtained in dark (laser mode having a dark spot at its center) and bright modes (laser mode having a peak of intensity at its center) in order to observe the sub-diffraction dimensions of the dark spot on the azimuthally polarized beam (doughnut-shaped light) (Dehez et al., 2013).

## Biochemical compartmentalization in the spine: a focus on Ca^2+^

Due to their small size, dendritic spines are well suited to the compartmentalization of biochemical and electrical signals. Indeed, biochemical signals, such as a buildup of intracellular Ca^2+^ after activation of glutamatergic receptors, have been shown to be compartmentalized in the spine head for several milliseconds (Yuste and Denk, 1995). The assumption that the spine morphology predicts biochemical compartmentalization is justified by a simplified compartmental model where the passive diffusional coupling of a molecule *x*, τ_*x*_, through the spine neck is given by,

(1) $$\tau_{x}=\frac{Vl}{DA}$$

where *V* is the volume of the spine, *l* is the neck length, *D* the diffusion coefficient of the molecule *x*, and *A* the cross-sectional area of the spine neck (*A* is defined as π*r*^2^, with *r* being the radius of the spine neck).

Recently, direct measurements of spine morphology in live tissue with STED imaging in combination with fluorescence recovery times after photobleaching (FRAP) (experimental τ, τ_*exp*_) of free diffusible fluorescence proteins (Tonnesen et al., 2014) or Alexa dyes (Takasaki and Sabatini, 2014; Tonnesen et al., 2014) indicated that τ_*exp*_ is determined by spine structure. As predicted by equation (1), τ_*exp*_ is negatively correlated with spine neck width, with small variations in neck diameter having significant effects on compartmentalization of fluorescent proteins and Alexa dyes (Takasaki and Sabatini, 2014; Tonnesen et al., 2014). Furthermore, it has been shown that τ_*exp*_ is positively correlated with spine neck length (strong linear correlation, *r* = 0.75, Takasaki and Sabatini, 2014; weak correlation, *r* = 0.42, Tonnesen et al., 2014) and spine head width (Tonnesen et al., 2014) (although see, Takasaki and Sabatini, 2014). In addition, using confocal microscopy and fluorescence loss in photobleaching (FLIP) it has been shown that τ_*exp*_ of membrane-bound fluorescent proteins is positively correlated with spine neck length and head size (Hugel et al., 2009). In agreement with these experimental findings, recent theoretical calculations using refined equations for the diffusion across the spine neck of a Brownian particle that is either inside the spine head or bound to its membrane suggest a strong dependency (negative correlation) between the diffusional coupling of a particle and (1) the spine neck length, and (2) the curvature of the connection between the spine head–neck (Holcman and Schuss, 2011). Hence, these experimental and theoretical results indicate that spine morphology predicts the compartmentalization of freely diffusible proteins, dyes and membrane-bound fluorescent proteins. Is this conclusion applicable for the spine–dendrite diffusion of ions and molecules such as Ca^2+^?

The development of Ca^2+^ imaging techniques such as 2P Ca^2+^ imaging (Denk et al., 1990) and the use of fluorescent Ca^2+^ indicators (Tsien, 1988) has opened up a means to explore neuronal activity with high spatial and temporal detail, providing a better understanding of the signaling pathways and function of subthreshold and suprathreshold spine Ca^2+^ signaling in synaptic transmission, storage and integration. Recently, the development of methods for data acquisition at high frame rates and low-excitation laser power has allowed researchers to perform 2P calcium imaging of dendritic spines *in vivo* (Chen et al., 2012b).

These advances have permitted imaging of the spatiotemporal calcium dynamics in single dendritic spines. For example, it has been reported that the decay time of Ca^2+^ in the spine head, τ_*Ca*_, has a positive correlation with the spine neck length (Majewska et al., 2000). In addition, combining electrical stimulation of dendritic spines with 2P monitoring of τ_*Ca*_ in the spine head and computer simulations has suggested that the amplitude of spine Ca^2+^ transients is positively correlated with the diffusional resistance of the spine neck (Grunditz et al., 2008), implying that the spine neck geometry can control the amplitude of the Ca^2+^ signal in the spine head as well as τ_*Ca*_, as predicted by simulations (Gold and Bear, 1994). Furthermore, it has been suggested that the spine head volume is negatively correlated with the amplitude of the glutamate uncaging-generated spine [Ca^2+^]_*i*_, but positively correlated with the [Ca^2+^]_*i*_ in the adjacent dendritic shaft (Noguchi et al., 2005). This suggests that spine neck and head morphologies are likely important determinants of the amplitude and diffusion of Ca^2+^ through the spine neck. In contrast, it has been shown using 2P uncaging of glutamate over single spines in combination with 2P calcium imaging that spine morphology cannot predict the amplitude of Ca^2+^ signals in spines (Sobczyk et al., 2005; Araya et al., 2006b, 2014). Moreover, a recent study using the same technical approach but also complemented by STED imaging showed the absence of a correlation between the peak Ca^2+^ amplitude and neck diameter or length (Takasaki and Sabatini, 2014). The reason for the discrepancy between these studies might be the fact that the spatiotemporal confinement of the Ca^2+^ signal is believed to rely not only on spine morphology but also on the characteristics of the synaptic input (Yuste and Denk, 1995; Sabatini et al., 2002), the variability and distribution of endogenous Ca^2+^ sensors (Baimbridge et al., 1992; Raghuram et al., 2012), the Ca^2+^ diffusion coefficient (Murthy et al., 2000), the presence and mobility of endogenous buffers (Gold and Bear, 1994; Murthy et al., 2000) and their Ca^2+^ binding ratios (Sabatini et al., 2002), as well as on active transport mechanisms, membrane potential and local spine activation of voltage-sensitive calcium channels (VSCCs) (Bloodgood and Sabatini, 2007), and the mechanisms for Ca^2+^ release from intracellular stores located within the spine head (Finch and Augustine, 1998; Takechi et al., 1998). However, the interplay between the various spine Ca^+2^ sensors and buffers (Raghuram et al., 2012), Ca^2+^ extrusion mechanisms (Ca^2+^ exit from the spine and/or sequestration into intracellular stores) (Yuste et al., 2000; Higley and Sabatini, 2012), activation of VSCCs (Bloodgood and Sabatini, 2007) and morphological spine features that explain Ca^2+^ compartmentalization and signaling in spines, as well as the compartmentalization of an array of other biochemical signals (Colgan and Yasuda, 2014; Sala and Segal, 2014), remain somewhat ill-defined.

### What are the pathways by which Ca^2+^ accumulates in the spine head of pyramidal neurons?

**Figure 2:**
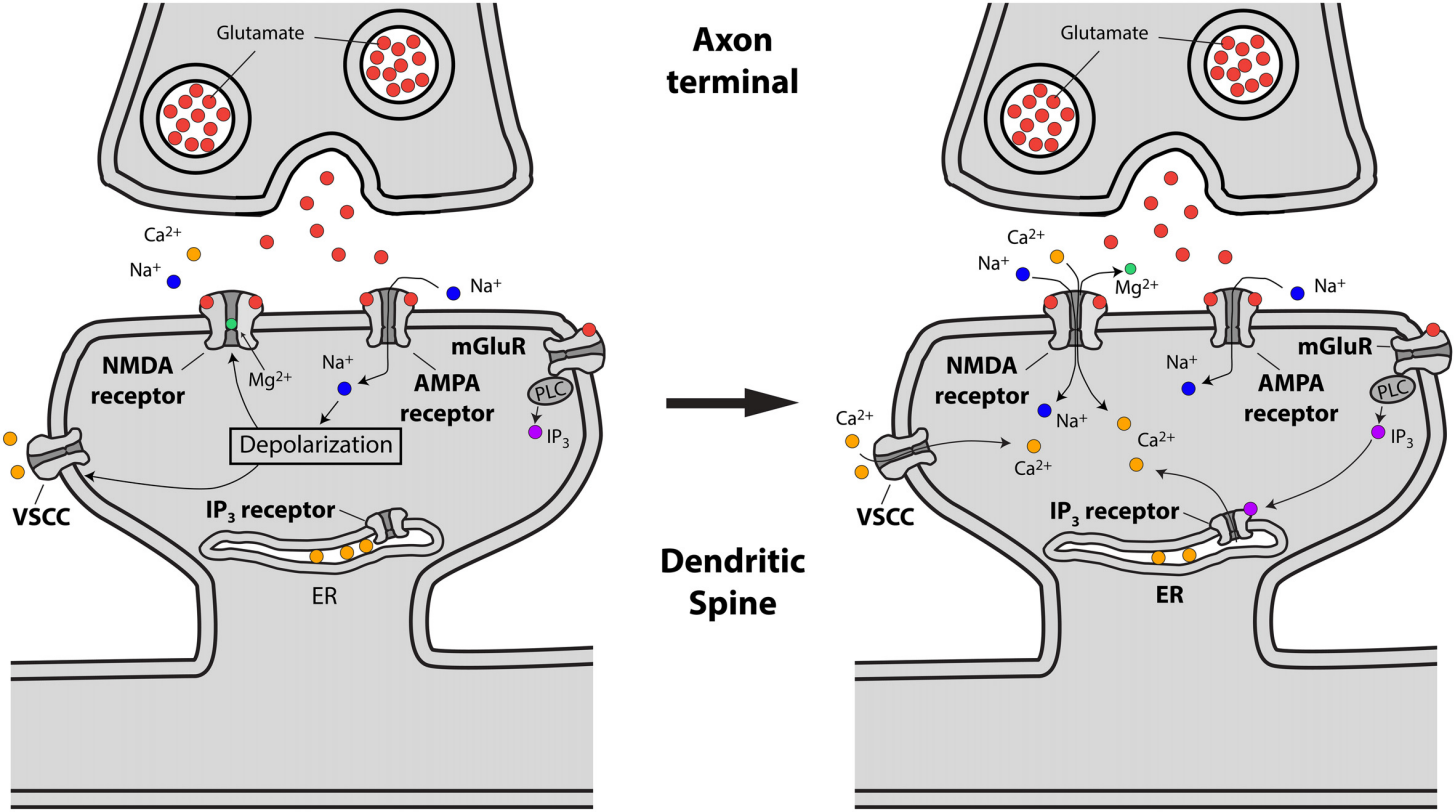
**Schematic representing excitatory synaptic transmission and the sources of Ca^2+^ accumulations at the spine head in pyramidal neurons**. **Left**, drawing showing how presynaptically released glutamate activates glutamate (AMPA, NMDA and mGluR) receptors leading to spine head depolarization. **Right**, spine depolarization will generate Ca^2+^ transients at the spine by removing the magnesium block from NMDA receptors, triggering the activation of voltage-sensitive Ca^2+^ channels (VSCCs) and the generation of second messengers like IP_3_ (for details see text).

The main pathways by which glutamate release from presynaptic terminals triggers a Ca^2+^ transient in the spine head are the following: First, the binding of glutamate to postsynaptic AMPA and NMDA glutamate receptors, followed by AMPA receptor-mediated membrane depolarization and Mg^2+^ unblock from the NMDA receptor, leads to the influx of both Na^+^ and Ca^2+^ into the spine head. Second, the depolarization provided by currents flowing through glutamate receptors has been suggested to lead to the activation of spine VSCCs (Bloodgood and Sabatini, 2007), which might provide an additional source of Ca^2+^ to the spine head. Third, Ca^2+^ can be released from internal stores via the metabotropic glutamate receptor (mGluR)-triggered production of inositol trisphosphate (IP_3_) in the spine head and the subsequent activation of IP_3_ receptors (Holbro et al., 2009; Oh et al., 2013) (Figure 2).

The activation of spine IP_3_ receptors and consequent release of Ca^2+^ from internal stores is required for long-term synaptic depression (LTD) (Holbro et al., 2009; Oh et al., 2013) and LTP (Raymond and Redman, 2006; de Sevilla and Buno, 2010). In addition, spine synapse-dependent Ca^2+^ transients are believed to be responsible for mediating LTP (Chittajallu et al., 1998; Lang et al., 2004; Matsuzaki et al., 2004; Harvey et al., 2008). The mechanisms through which this occurs include the activation of calcium/calmodulin-dependent protein kinase II (CaMKII) and modification of the AMPA receptor conductance, plasma membrane insertion of AMPA receptors and recruitment of surface AMPA receptors into the synapse (Lisman et al., 2012), modifications to the internalization of spine voltage-gated channels (Kim et al., 2007), and alterations in spine actin dynamics (Fortin et al., 2012; Sala and Segal, 2014). The generation of LTP in individual spines by repetitive 2P uncaging of glutamate has been shown to produce increases in spine head volume that are associated with a Ca^2+^- and calmodulin-dependent actin reorganization process (Matsuzaki et al., 2004). In addition, studies in which LTD was triggered using low-frequency electrical stimulation or 2P uncaging of glutamate have demonstrated that spine heads may shrink in volume (Zhou et al., 2004; Oh et al., 2013; Wiegert and Oertner, 2013) via a mechanism dependent on mGluRs, IP_3_Rs and Ca^2+^ (Oh et al., 2013), or that spines may even retract completely (Nagerl et al., 2004; Wiegert and Oertner, 2013). The activity-dependent structural spine changes observed after LTP and LTD are thought to contribute to the experience-dependent brain changes associated with learning and memory (Lynch, 2004; Holtmaat and Svoboda, 2009; Kasai et al., 2010). Recently, it has been demonstrated that changes in synaptic strength via LTP and LTD are linked with memory formation (Nabavi et al., 2014), suggesting that spines are indeed the functional unit of learning and memory.

In both LTP and LTD, the activation of NMDA receptors and the subsequent increase in spine Ca^2+^ has been suggested to be differentially regulated by the recruitment of separate molecular pathways (Malenka and Bear, 2004). These separate pathways are initiated by the different spatiotemporal Ca^2+^ signals generated by protocols that trigger LTP [high-frequency stimulation (HFS)] or LTD [low-frequency stimulation (LFS)], with LTP being triggered by fast and large spine Ca^2+^ signals within the spine head and LTD triggered by Ca^2+^ signals with differing magnitude and/or duration (i.e., small Ca^2+^ signals) (Malenka and Bear, 2004). Furthermore, NMDA receptor activation and increases in spine Ca^2+^ are required for a process known as spike-timing dependent plasticity (STDP). STDP is a variation of LTP and LTD that has been described in pyramidal cells and involves the pairing of pre- and postsynaptic action potentials (Magee and Johnston, 1997; Markram et al., 1997; Bi and Poo, 1998; Debanne et al., 1998; Zhang et al., 1998). In this process, the relative timing of pre- and postsynaptic action potentials determines the polarity and magnitude of the change in synaptic strength (Zhang et al., 1998). These timing rules are altered in mouse models of Rett and Fragile-X syndromes, two X-linked neurological disorders (Desai et al., 2006; Meredith et al., 2007). Thus, STDP is an important model for understanding learning and memory and is of key importance for understanding developmental and neurodegenerative disorders in which spine structure is impaired (Fiala et al., 2002). Many questions regarding the induction paradigms and molecular cascades responsible for the generation of STDP in the spines of pyramidal neurons remain to be determined.

The fact that spines exist in a variety of head and neck morphologies (Spacek and Harris, 1998; Sorra and Harris, 2000; Arellano et al., 2007b; Takasaki and Sabatini, 2014; Tonnesen et al., 2014) and that the spine neck seems to be important in controlling the amplitude and diffusion of Ca^2+^ out of the spine head (Gold and Bear, 1994; Majewska et al., 2000; Noguchi et al., 2005; Grunditz et al., 2008) is suggestive of a process in which spine morphology not only determines the amplitude and spatiotemporal confinement of Ca^2+^ in the spine head, but also the generation of plasticity. In agreement with this notion, recent modeling studies showed that the relationship between Ca^2+^ influx and spine head morphology is key for determining synaptic stability (O'Donnell et al., 2011). Another important determinant of Ca^2+^ influx in the spine head might be related to the electrical properties of spines (see below) (Grunditz et al., 2008; Bloodgood et al., 2009), in particular how variations in spine neck morphology affect neck resistance (*R*_N_, see below) and synaptic amplification and ion influx in the spine head. Although there is evidence linking spine geometry with the compartmentalization of Ca^2+^ signals in the spine head, how spine geometry affects the molecular machinery and synaptic efficacy during plasticity remains ill-defined. The plethora of functions exerted by Ca^2+^ in the spine is most readily explained by the ability of synaptic inputs and backpropagating action potentials to generate Ca^2+^ signals with different amplitude, kinetics, and spatiotemporal confinement (Sabatini et al., 2002). These features enable the differential activation of signaling pathways (Malenka and Bear, 2004) and lead to structural rearrangements, thereby modifying the close relationship between spine structure and function.

## Electrical compartmentalization of the spine

### Role in synaptic transmission

Theoretical studies looking at the electrical behavior of spines have suggested that their electrical properties may result in the generation of large excitatory post-synaptic potentials (EPSPs) at the spine head (Jack et al., 1975; Segev and Rall, 1988) that are sufficient to activate spine voltage-gated channels and thus modify synaptic efficacy (Miller et al., 1985; Perkel and Perkel, 1985; Shepherd et al., 1985; Segev and Rall, 1988). These predictions are based on Ohm's law (*V* = *I* * *R*, where *V* is the potential, *R* the resistance and *I* the current) and can be understood by considering the spine in the form of an equivalent circuit, consisting of a spine head connected to the dendrite by a slender neck. Each of these membrane compartments can be represented by a resistance–capacitance (RC) circuit (Figure 1C). Hence, a useful simplified model to study the generation and propagation of excitatory postsynaptic potentials from the spine head to the dendritic shaft considers the capacitance of the spine head (*C*_*m*(*h*)_), neck (*C*_*m*(*N*)_) and dendrite (*C*_*m*(*d*)_); their membrane resistance (*R*_*m*(*h*)_, *R*_*m*(*N*)_, *R*_*m*(*d*)_) and the synaptic conductance at the spine head (*G_syn_*), as well as other conductances at the spine head, neck (*G_N_*), and dendrite (*G_d_*). In addition, the overall spine neck resistance (*R_N_*, see below) and dendritic resistance (*R_d_*) are important factors in controlling the spread of synaptic potentials (Figure 1C). Since the spine head and neck have a small surface area their capacitance is negligible. Hence, in this model, the excitatory postsynaptic potential at the spine head (*EPSP_spHead_*) is represented by

(2) $$EPSP_{spHead}\approx I_{syn}\;*\;(R_{N}+Z)$$

Where *I_syn_* is the synaptic current, *R_N_* the spine neck resistance, and *Z* the dendritic impedance (a property dependent on the resistance and capacitance). Based on dendritic dimensions, *Z* is expected to be both much smaller than, and not as easily modified as, *R_N_*. In addition, *R_N_* is determined by

(3) $$R_{N}=4\rho l/\pi d^{2}$$

Where *l* is the spine neck length, *d* its diameter and ρ the axial resistivity (Koch, 2004). It is important to note that although it is possible to experimentally measure spine *l* and *d* (Table 1), the current technologies prevents us measuring the spine neck ρ, hence calculations of *R_N_* using arbitrary values of spine neck ρ (Tonnesen et al., 2014) might not be adequate. Thus, the amplitude of the EPSP at the spine head (*EPSP_spHead_*) and the degree of passive spine voltage amplification may be drastically modified by changes in *R_N_*, as determined by the neck length *l*, diameter *d* and axial resistivity ρ (Koch, 2004). In addition, these predictions indicate that a high *R_N_* will generate large and fast EPSPs at the spine head, which has been suggested to diminish the location-dependent variability of spine potentials (Gulledge et al., 2012) that would otherwise be expected if inputs impinged directly onto the dendritic shaft (Rinzel and Rall, 1974). Furthermore, theoretical studies have proposed that the slender spine neck has an *R_N_* high enough to significantly attenuate the synaptic potential between the spine head and its parent dendrite, therefore affecting synaptic efficacy (Chang, 1952; Llinás and Hillman, 1969; Diamond et al., 1970; Rall, 1974; Jack et al., 1975; Koch and Poggio, 1983a; Koch et al., 1983; Segev and Rall, 1988; Koch, 2004).

#### Is *R_N_* sufficient to control synaptic weight and thereby modify somatic EPSPs?

**Figure 3:**
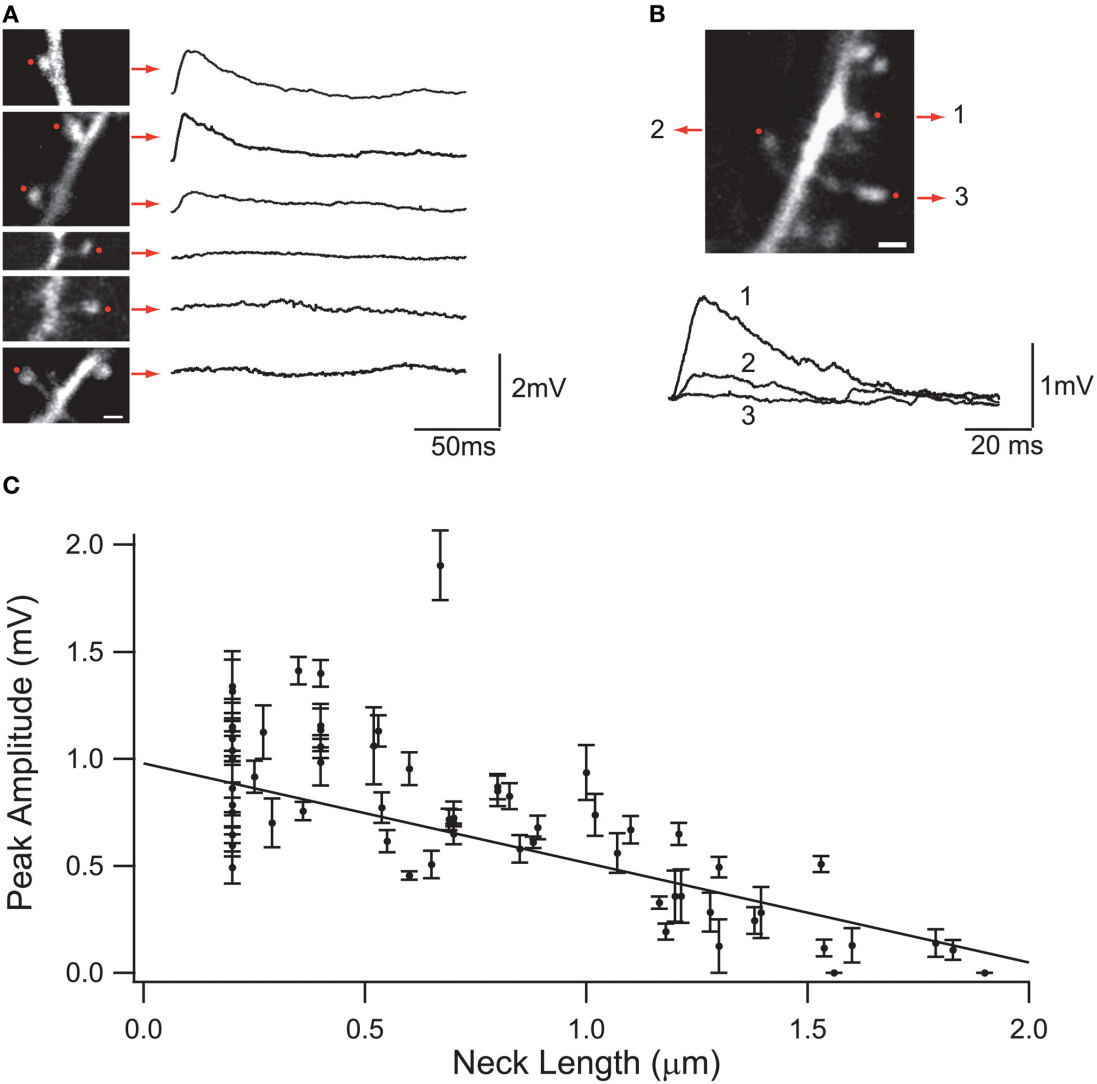
**Effect of the spine neck on spine uncaging potentials. (A)** Examples of two-photon glutamate uncaging potentials in spines with different neck lengths. Red dots indicate the site of uncaging, and traces correspond to averages of 10 uncaging potentials from each spine. **(B)** Three neighboring spines with different neck lengths. Note the different uncaging potentials generated at the soma of the neuron. **(C)** Plot of the uncaging potential peak amplitude versus neck length. Line is the linear regression of the data with a weighted fit. Standard errors are provided for each point (Figure taken with permission from Araya et al., 2006b).

Synaptic inputs in individual spines can be mimicked via 2P uncaging of caged glutamate (Matsuzaki et al., 2001; Araya et al., 2006a, 2014; Bloodgood and Sabatini, 2007; Harvey et al., 2008; Harnett et al., 2012). Using this technique, it has been shown in cortical pyramidal neurons that the amplitude of the uncaging evoked spine potentials recorded at the soma are inversely proportional to the length of the spine neck (Araya et al., 2006b; Richardson et al., 2009) (Figure 3). In addition, a recent report, using STED-2P imaging and 2P uncaging of glutamate in individual spines of CA1 hippocampal pyramidal neurons, showed an inverse correlation between spine neck length and uncaging potentials recorded at the soma, but with a weaker correlation (*p* = 0.09) than that found in cortical pyramidal neurons (Takasaki and Sabatini, 2014). This apparent discrepancy might depend on dissimilarities between cortical and hippocampal pyramidal spines, or simply because the data from Takasaki and Sabatini (2014) explored spines with a narrower range of neck lengths (~0.2–1.2 μm) than that from Araya et al. (~ 0.2–2 μm) (Araya et al., 2006b). Furthermore, we recently used minimal synaptic stimulation of identified spines and confirmed that EPSP amplitudes are indeed inversely correlated with spine neck lengths, although no significant changes occur in the amplitude of the spine Ca^2+^ response, as measured using 2-photon glutamate uncaging (Araya et al., 2014). This is similar to the reported lack of correlation between spine Ca^2+^ and neck length when using glutamate uncaging at single spines together with 2P STED imaging (Takasaki and Sabatini, 2014).

**Figure 4:**
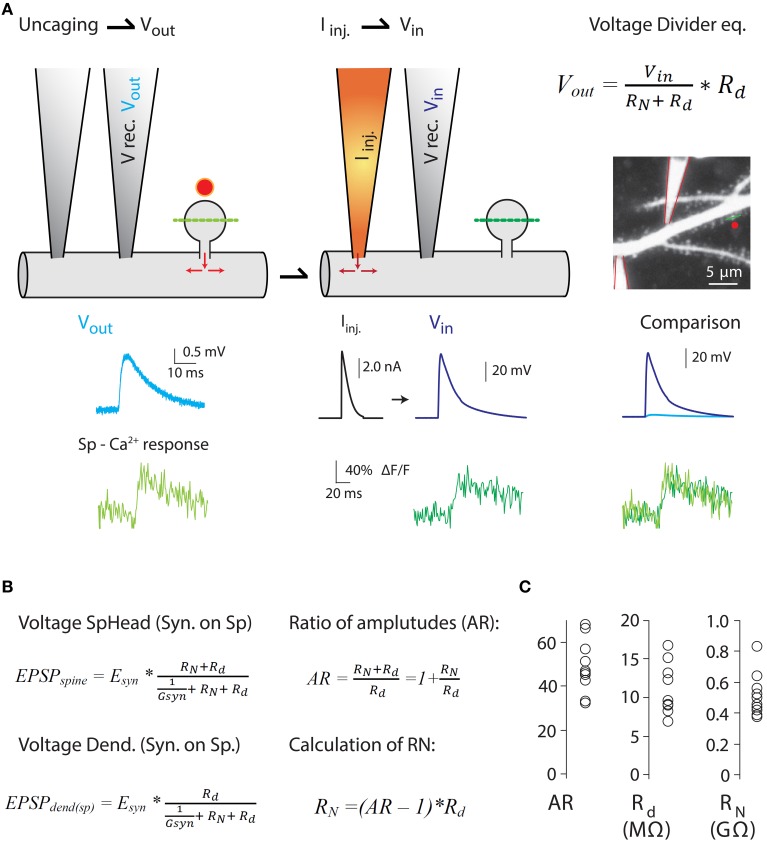
**Measurements of the ratio of spine-to-dendrite voltage amplitude, to estimate *R_N_* using the voltage divider equation**. Harnett et al. (2012) estimated *R_N_* by combining two-photon Ca^2+^ imaging and glutamate uncaging with dual dendritic patch-clamp current injection and voltage recording from hippocampal CA1 pyramidal neurons in acute slices. **(A)** Uncaging potential [light blue trace in **(A)**] was produced by uncaging onto a single spine and recording in the dendrite (V rec. V_*out*_) while measuring spine head Ca^2+^ responses (Sp-Ca^2+^ response, light green trace) mediated exclusively by voltage-sensitive Ca^2+^ channels (VSCC) (see Harnett et al., 2012 for details). Next, current injection (I_*inj*._) into the dendrite was performed to depolarize the spine to a level that triggers Ca^2+^ responses in the spine head (dark green traces) similar to the ones produced by glutamate uncaging (V_*in*_ in voltage divider equation. Also see comparison of V_*in*_ and V_*out*_ spine head Ca^2+^ responses). Assuming a lack of voltage attenuation from the dendrite to the spine, the uncaging potential and the voltage generated by I_*inj*_ provide a good estimate of the spine head potentials (V_*in*_). Two-photon image of a dendrite patched with two patch electrodes, and the voltage and calcium traces were taken from Harnett et al. (2012). **(B)** The amount of electrical compartmentalization produced by the spine can be measured as the amplitude ratio (AR) of the voltage at the spine head when an input impinges on the spine (*EPSP_spine_*), to the voltage at the dendrite when the synapse impinges on the spine (*EPSP*_*dend*(*sp*)_). **(C)** Calculation of *R_N_* was obtained by the equation depicted in **(B)**. Modified with permission from Harnett et al. (2012). See Harnett et al. (2012) for details.

An intuitive way of capturing the essence of the relation between somatic EPSP and *R_N_* can be obtained by using the voltage divider equation for inputs impinging onto a spine or dendrite (Johnston and Wu, 1995)

(4) $$EPSP_{dend(sp)}=E_{syn}\;*\;\frac{R_{d}}{\frac{1}{Gsyn}+R_{N}+R_{d}}$$

(5) $$EPSP_{dend(dend)}=E_{syn}\;*\;\frac{R_{d}}{\frac{1}{Gsyn}+R_{d}}$$

where *EPSP*_*dend*(*sp*)_ is the amplitude of the voltage generated in the dendrite at the place where the spine is attached, when the synapse occurs at the spine head, and *EPSP*_*dend*(*dend*)_ is the amplitude of the voltage in the dendrite when the synapse is located in the dendrite. *R_d_* represents the input resistance of the dendrite at the place where the spine is attached to the dendrite; *G_syn_*, the synaptic conductance; *R_N_*, the spine neck resistance; and *E_syn_*, the synaptic reversal potential. Using equations (4) and (5) we can arrive at a simplified formula that depicts the relative effectiveness of a synapse on a spine compared with one directed onto the dendrite

(6) $$\frac{EPSP_{dend(sp)}}{EPSP_{dend(dend)}}=\frac{1}{1+P}$$

in which *P* is the product of *G_syn_* and *R_N_*. For simplicity, I assume a negligible value of *R_d_*. This assumption relies on the fact that the cross sectional area (*A*) of a dendrite is much larger than that of the spine neck, and hence *R_d_* is much smaller than *R_N_* (see Figure 4C for estimated values of *R_N_* and *R_d_*). Second, the assumption is made because the goal of the formulation is to evaluate the effect of the spine neck on synaptic transmission from the spine head to the dendrite through the neck. This fit reveals that provided *P* is much less than 1, there should be no effect of *R_N_* on dendritic or somatic EPSPs, and a synapse onto a spine could be approximated as a constant current source of amplitude *G_syn_* * *E_syn_*. It is important to note that due to the differential input resistance of the spine head and dendrite, the voltage at the spine head is not the same as the voltage observed in the parent dendrite, with an attenuation factor given by

(7) $$\frac{EPSP_{dend(sp)}}{EPSP_{dend(dend)}}=\frac{R_{d}}{R_{N}+R_{d}}$$

At the other extreme, if *P* >> 1, then the EPSP at the spine head will start to reach *E_syn_* and the spine will act as a voltage source, where changes in spine neck length will affect the amount of current entering the spine, and as a consequence the somatic EPSP and the neck length *l* should be reciprocally related (Koch and Poggio, 1983b) (Figure 3). The rate of somatic voltage attenuation with neck length, obtained from the slope of the linear fit to the experimental data from spines from neocortical pyramidal neurons (Araya et al., 2006b, 2014), implies under these assumptions that *P* ~ 1, therefore suggesting that *R_N_* is appreciable.

#### What is the value of *G_syn_* for individual pyramidal neuron spines?

Unfortunately, this value cannot be measured directly at the spine head. However, indirect measurements of AMPA and NMDA receptor unitary synaptic conductances and their content per spine can prove useful when trying to estimate *G_syn_* in single dendritic spines. Indeed, non-stationary fluctuation analysis from EPSCs obtained by 2P uncaging of glutamate over individual CA1 pyramidal neuron spines gave an estimated average AMPA receptor unitary current of 0.6 pA and an AMPA receptor number per spine of 46–147 (mean 82) (Matsuzaki et al., 2001). Thus, the conductance of a single AMPA channel (γ) located in a spine can be calculated by dividing the unitary current (0.6 pA) by the driving force at a resting membrane potential of the recorded cells (clamped at −65 mV in Matsuzaki et al., 2001). This gives a value of γ = 9.2 pS, similar to the calculated AMPA receptor γ value of ~8 pS obtained from non-stationary fluctuation analyses of synaptic responses in CA1 pyramidal neurons (Benke et al., 1998). Although there was some variability in the unitary synaptic conductance reported in both articles, most of the inputs triggered small unitary synaptic conductances [(Benke et al., 1998), mean value for γ of 7.7 ± 0.7 pS; (Matsuzaki et al., 2001), mean AMPA unitary current of 0.6 ± 0.1 (or 9 pS)]. By multiplying the number of AMPA receptors per spine by γ, the total estimated AMPA receptor dependent synaptic conductance per spine is ~0.4–1.4 nS (mean 0.8 nS) for a CA1 pyramidal neuron. Given these estimated AMPA-only *G_syn_* values, the addition of NMDA receptors and active conductances could easily produce *G_syn_* values of ≥1 nS. At such *G_syn_* values and with the simplified assumption of *P* ~ 1, (this) supports the somatic voltage attenuation with spine neck length observed experimentally (Araya et al., 2006b, 2014), and implies *R_N_* values of ~1 GΩ.

Furthermore, as predicted by modeling studies (Miller et al., 1985), voltage-gated ion channels in the spine are recruited independently of those in the dendritic shaft (Araya et al., 2007; Bloodgood et al., 2009), thereby affecting synaptic efficacy (Miller et al., 1985; Araya et al., 2007; Allen et al., 2011). These data suggest that spines have an appreciable *R_N_* that allows them to act as electrical compartments with active conductances, but it is unclear if the experimentally observed correlation between spine neck length and EPSP amplitude recorded at the soma can be explained entirely or only partly by the passive attenuation of synaptic potentials through the spine neck. The explanation as to why this question remains unresolved resides in the experimental limitations of current electrophysiological techniques, which are incapable of directly measuring *R_N_* or absolute spine voltage responses. However, different experimental strategies have been implemented to estimate *R_N_*, providing values ranging from just a few MΩ (Svoboda et al., 1996; Grunditz et al., 2008; Palmer and Stuart, 2009; Tonnesen et al., 2014) up to ~500 MΩ (Palmer and Stuart, 2009; Harnett et al., 2012) or 1 GΩ (Bloodgood and Sabatini, 2005; Grunditz et al., 2008). A recent study by Magee and colleagues used a clever experimental design in which 2P Ca^2+^ imaging, glutamate uncaging, and dual dendritic patch-clamp recording and current injection were combined to estimate the *R_N_* of spines belonging to CA1 pyramidal neurons using the voltage divider equation (Figure 4) (Harnett et al., 2012). Their data showed that all spines they analyzed had high *R_N_* (spines with an apparent short neck), with an average value of ~500 MΩ—sufficient to amplify spine potentials to ~25 mV for an average unitary event and to enhance input cooperativity (Harnett et al., 2012). Their experimental design and the variables recorded in order to estimate *R_N_* can be seen in Figure 4.

#### Are *R_N_* values of ~500 MΩ sufficient to influence somatic epsp amplitude and thereby provide a mechanism for controlling synaptic efficacy?

Experimental evidence therefore indicates that relatively high values of *R_N_* can lead to amplification of synaptic inputs at the spine head. The question then arises as to whether similar *R_N_* values may be sufficient to influence somatic EPSP amplitude. In this regard, numerical simulations of spines using a *G_syn_* of 500 pS have shown that an *R_N_* of 500 MΩ can cause a reduction in somatic EPSP amplitude of only ~15% (Palmer and Stuart, 2009). In addition, our own simulations using morphologically realistic multi-compartmental models to explore the passive spine properties and *R_N_* values required to reproduce the experimentally obtained inverse correlation between neck length and somatic EPSP amplitude (Araya et al., 2006b, 2014; Richardson et al., 2009; Vogels et al., 2009) gave us *R_N_* values that are at odds with previous *R_N_* estimates (Harnett et al., 2012). Thus, it is unlikely that *R_N_* values of ~500 MΩ can significantly influence somatic EPSP amplitude and explain the neck length control of somatic EPSP amplitude. Instead, the inverse correlation we observed may result from a combination of passive (e.g., through a reduction in driving force for the synaptic current entering the spine head with increasing *R_N_*, see above) and active spine mechanisms (e.g., active dampening of EPSPs by the engagement of spine voltage-gated potassium channels) and/or the differential control of AMPA receptor content between spines of different neck lengths.

Recently, by inducing LTP with an STDP protocol in which 2P uncaging of glutamate over individual spines was paired with bAPs (Tanaka et al., 2008), it was demonstrated that activated spines undergo activity-dependent structural changes (Tanaka et al., 2008; Araya et al., 2014), with long- and short-necked spines experiencing a rapid shrinkage in spine neck length that correlated with an increase in the somatically recorded uncaging potential (Araya et al., 2014). These results could provide an explanation as to why pyramidal neuron dendrites are covered with long-necked spines, providing a reservoir of connectivity that can be called into action upon activity, without the need to rewire the neuronal network.

To better understand the electrical properties of spines and the implications for synaptic transmission and plasticity, further experiments devoted to understanding the passive and active mechanisms controlling synaptic efficacy and synaptic amplification at the spine head are required. In addition, it seems likely that to achieve a full understanding of these issues, strategies that allow neural electrode size to be substantially reduced—capable of recording directly from the spine head and parent dendrite—as well as proper optical voltage-sensing probes must be developed to directly measure absolute spine potentials, neck resistivity ρ and *R_N_*.

### Role of spines in synaptic integration and plasticity: clustered vs distributed connectivity

Two fundamental questions in neuroscience are (1) what is the spatiotemporal pattern of the multitude of excitatory inputs impinging on a pyramidal neuron? and (2) how can structural and molecular remodeling at the synaptic level support synaptic plasticity, modify the strength of individual synapses and change the input/output properties of a pyramidal neuron?

Two main models exist for the possible distribution of synaptic inputs: clustered, in which synchronous or asynchronous synaptic inputs arrive at a spatially restricted zone in the dendrites of the postsynaptic neuron; and distributed, in which inputs are spread along the dendritic arbor of the postsynaptic neuron. Related to this are the concepts of random and structured connectivity. Random connectivity naturally implies that connections between two neurons occur by chance, and inputs should therefore be distributed approximately randomly over the dendritic tree (Peters and Feldman, 1976). Structured connectivity, however, allows for clustered inputs (although inputs may also be “structured” to achieve distributed inputs), which implies that there is a choice by the presynaptic neuron as to where to form a synaptic connection. Clearly, the fact that excitatory inputs are directed to spines rather than shaft locations implies that connectivity is not entirely random. The level of randomness within a structured connectivity paradigm could then be defined at the next level—how excitatory inputs, directed to spines, are placed along the dendritic tree of the postsynaptic pyramidal neuron; are they distributed or clustered? What are the functional consequences and computational power conferred by having inputs distributed or clustered in the dendrites? Single-synapse resolution reconstructions of axons and dendrites from connected pairs of layer V thick-tufted pyramidal neurons of the somatosensory cortex have suggested that axons touch all neighboring dendrites in a distributed manner without any bias (Kalisman et al., 2005). Consistent with this, a more recent study from the Konnerth laboratory using high speed *in vivo* 2P imaging of dendrites and electrophysiological recordings from Layer II pyramidal neurons revealed that orientation-tuned neurons received spatially distributed synaptic inputs to generate their characteristic firing pattern (Jia et al., 2010). Moreover, in a follow-up paper, the same group performed *in vivo* imaging of spine activity in the dendrites of Layer II pyramidal neurons and demonstrated that a sound stimulus activated spines that were broadly distributed on basal and apical dendrites (Chen et al., 2011).

However, not all studies have found such spatially distributed connectivity, instead obtaining evidence for clustered connectivity. Recently, experiments performed in neuronal hippocampal slice cultures and in Layer II/III barrel cortex pyramidal neurons *in vivo* showed that activity frequently occurred in neighboring spines (Takahashi et al., 2012), a finding that was also observed during the development of hippocampal pyramidal neurons (Kleindienst et al., 2011, for review see DeBello et al., 2014).

#### But how would distributed or clustered connectivity affect neuronal output?

The first to propose that the mode of integration of coincident synaptic inputs impinging directly on the dendrite will depend greatly upon their dendritic location was Rall (1964), who stated:

“*These results show that, although the departure from linearity* [linearity meaning the arithmetic sum of the synaptic events] *can become quite large when perturbations are superimposed upon the same compartment, the departure from linearity can be surprisingly small when brief perturbations occur in separate portions of the dendritic periphery*.”

**Figure 5:**
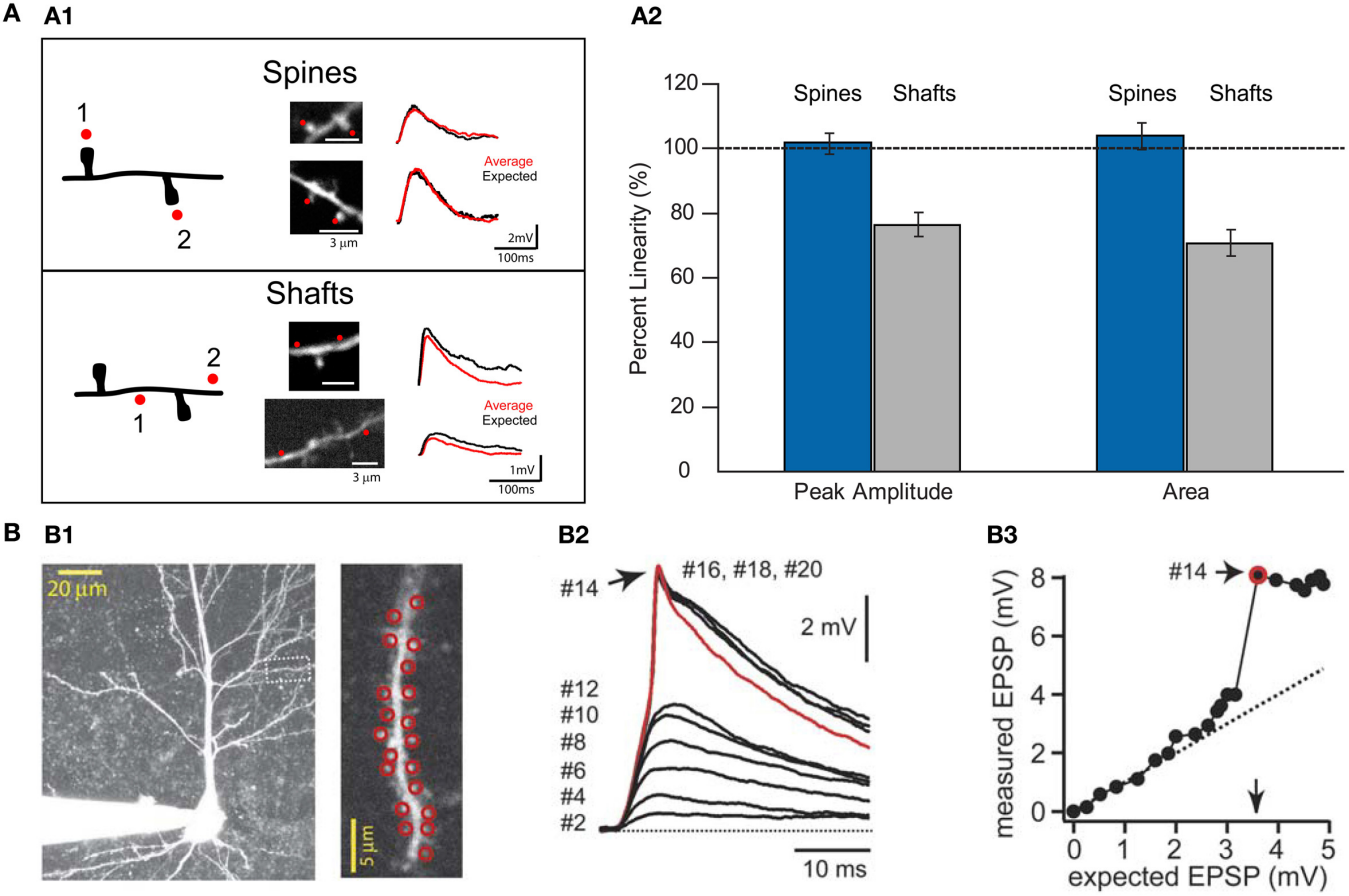
**Summation of excitatory uncaging potentials on spines and dendritic shafts. (A1)** Left, drawing of a dendrite from a layer V pyramidal cell showing the protocol for testing summation in spines and shafts. Red dots indicate the sites of uncaging in spines or shafts. Middle, two-photon images showing the uncaging locations in spines or shafts (red dots). Right, voltage responses were recorded with a patch electrode in current-clamp configuration. Two-photon uncaging of glutamate was performed first at each spine or shaft location (1 or 2) and then in either both spines together or in both shaft locations (1 + 2). Summation in spines: Red trace corresponds to an average of 10 depolarizations caused by uncaging over the two spines, and black traces correspond to the expected algebraic (linear) sum of the individual events of each spine. Summation in shafts: Data are presented as for spines. Note how the average uncaging response when spines are activated is close to the expected value. However, when inputs impinge on shaft locations, the integration is sublinear (Image modified from Araya et al., 2006a). **(A2)** Summary of results from Araya et al. (2006a). Data are presented as averages ± s.e.m. **(B)** Data taken with permission from Losonczy and Magee (2006). **(B1)** Two-photon image stack from a CA1 pyramidal neuron. Inset, red circles indicate the site of uncaging in spines—up to 20 spines in this example. **(B2)** Two-photon uncaging potentials evoked at a 0.1 ms interval, ranging from 2 to 20 activated spines. **(B3)** Input/output plot for the experiment. Note how inputs onto spines integrate linearly before additional inputs generate a dendritic spike.

**Figure 6:**
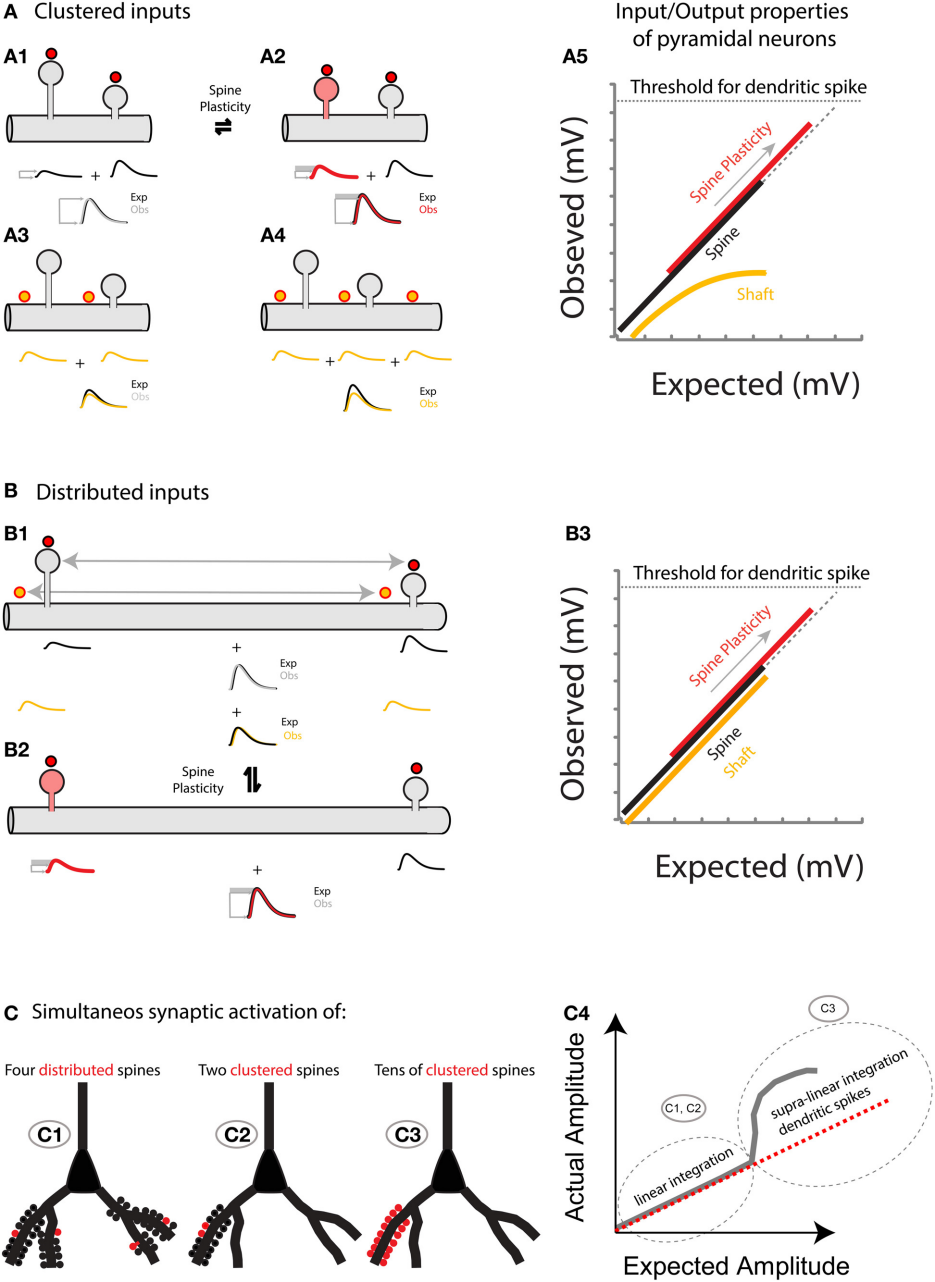
**(A)** Spatially clustered inputs: Excitatory inputs directed to clustered spines add linearly (**A1**, compare the expected algebraic (linear) sum (Exp., black trace) of the individual events of each spine with the observed response after simultaneous activation of all spines (Obs., gray trace)) before the generation of a dendritic spike (**A5**, dotted line indicates the threshold for triggering a dendritic spike). In contrast, excitatory inputs directed to clustered shaft locations will shunt each other (**A3** for 2 inputs, and **A4** for 3 inputs). Note that more shunting is expected if more clustered inputs are directed to the shaft (compare Exp. (black trace) vs. Obs. (yellow trace) in **A3**,**A4**). **A5**, Plot of the observed vs. expected amplitude (mV) for uncaging events in spines (black) or shafts (yellow) along the dendrite of layer 5 pyramidal neurons. Plasticity (for simplicity I only focus here on neck plasticity as reported in Araya et al., 2014): Spine-STDP will generate a significant change in the neck length (and probably a conductance change) of the stimulated spine (**A2**, red spine) with a concomitant change in synaptic weight (red trace, gray box shows the amplitude change from control). Single spine-STDP will increase the input/output gain (**A5**, red trace and arrow indicating the change in gain from the control (black) trace) of the neuron without affecting the linear integration of subthreshold excitatory inputs. **(B)** Spatially distributed inputs: Distributed excitatory inputs directed to spine (red dots) or shaft locations (yellow dots) will integrate linearly (**B1**, compare Exp. Vs. Obs.) by preventing large variations in the input impedance of the dendrite, thus avoiding shunting interactions that would otherwise be expected if clustered inputs are directed to the dendritic shaft (**A3**,**A4**). **B3** same as **A5** but with distributed inputs onto spine and shaft locations. **(C)** Representation of the summation of excitatory inputs directed to spines of a pyramidal neuron. The simultaneous synaptic activation of a few distributed (**C1**) or clustered (**C2**) spines (red) would trigger a voltage response that matches the arithmetic linear sum of each spine's voltage contribution. If tens of spines are activated simultaneously within the same branch (**C3**), then a supralinear response, or dendritic spike, will be generated.

Theoretical predictions and experimental studies—in which numerous spines are activated almost simultaneously by means of 2P uncaging of glutamate—have shown that the activation of a small number of neighboring spines results in linear integration (Poirazi et al., 2003; Araya et al., 2006a; Losonczy and Magee, 2006; Gomez Gonzalez et al., 2011) (Figure 5), but that the addition of clustered excitatory inputs causes a threshold to be reached for the generation of a non-linear, suprathreshold integrative voltage response—or spike—generated in the dendrites (Gasparini and Magee, 2006; Losonczy and Magee, 2006; Harnett et al., 2012) (Figure 5). Indeed, it is well known that neocortical pyramidal neuron dendrites are capable of triggering sodium, calcium, and NMDA spikes (Larkum and Nevian, 2008; Major et al., 2008; Larkum et al., 2009; Murayama et al., 2009; Polsky et al., 2009). Thus, the presence of clustered inputs and the generation of non-linear responses can increase the computational power of dendrites (Losonczy et al., 2008), for example by changing the threshold for LTP at local (to the input) spines (Harvey et al., 2008) and selectively enhancing excitability in dendrites (Losonczy et al., 2008), as well as serving as a mechanism to overcome the distance dependence of synaptic efficacy (Williams and Stuart, 2003; Spruston, 2008). However, in a fully distributed network, this level of structural and functional dendritic fitness would not be necessary for neuronal and network computations, and synaptic transmission and storage will mainly be controlled by the biochemical and electrical properties of spines (Figure 6).

In conclusion, significant evidence has accumulated in favor of both the distributed and clustered input hypotheses. However, whether these are mutually exclusive has not yet been resolved, and the divergent results may be due to different regimes being recruited under different circumstances. In addition, how dendrites and spines transform different spatiotemporal input sequences into different output patterns, and how these inputs can trigger changes in synapse strength and affect experience-dependent learning, remain ill-defined.

## A hypothesis for the *raison d'etre* of dendritic spines

In 1969, Llinás and Hillman (1969) suggested that if synaptic inputs are directed simultaneously to dendritic spines with high *R_N_*, the synapses would be converted into a near constant current system that protects the length constant of the dendrite by preventing variations in input resistance, producing a more linear summation of synaptic potentials in spines that belong to the same dendritic compartment. In addition, they predicted that if synaptic inputs are instead directed toward the dendritic shaft, the inputs would summate in a more non-linear fashion (Llinás and Hillman, 1969). Indeed, as pointed out before, experiments using nearly simultaneous 2P uncaging of glutamate to activate 2–3 (Araya et al., 2006b), 7–10 (Gasparini and Magee, 2006) or up to ~20 spines (Losonczy and Magee, 2006) located in the same dendritic compartment (covering <20 μm of the dendrite) demonstrated that excitatory inputs onto spines integrate linearly before additional inputs generate a dendritic spike (Losonczy and Magee, 2006) (Figure 5B), whereas inputs delivered to the same compartment but onto the dendritic shaft integrate sublinearly (Araya et al., 2006a) (Figure 5A), most likely due to a local decrease in driving force or shunting interactions between the excitatory inputs, as proposed by Llinás and Hillman (1969) (Figures 5, 6). These results and predictions suggest that the departure from linearity in pyramidal neurons (and other neurons) should be small or negligible when excitatory inputs are directed to separate portions of the dendritic shaft (Rall, 1974) (Figure 6). Indeed, the degree of linear summation between converging EPSPs on fast spiking (FS) cells correlates with the distance between the nearest neighboring synapses impinging on the dendritic shaft of FS cells (Tamas et al., 2002).

### Is the observed subthreshold linear summation of synchronous excitatory inputs protected by the spine *R_N_*?

Numerical simulations have indicated that two neighboring, simultaneously active spine synapses could reproduce the linear integration observed experimentally (Araya et al., 2006a) by building spines with *R_N_* of 600 MΩ (Grunditz et al., 2008), similar to the *R_N_* values calculated from the spines of CA1 pyramidal neurons (Harnett et al., 2012). In addition, these simulations showed that the same synapses can experience sublinear integration when *R_N_* is lowered to 100 MΩ (Grunditz et al., 2008), resembling the experimentally observed sublinear integration when neighboring shaft locations were activated (Araya et al., 2006a) (Figure 5A). In these simulations the high spine *R_N_*-dependent linear summation of excitatory inputs depends on electrical amplification and the recruitment of voltage-gated channels at the spine head (Grunditz et al., 2008). These experimental results (Araya et al., 2006a; Gasparini and Magee, 2006; Losonczy and Magee, 2006), together with modeling predictions (Grunditz et al., 2008), imply that most if not all spines with short- and long necks act as electrical compartments, having *R_N_* values that exceed the critical threshold for promoting the linear integration of excitatory inputs (Figures 5, 6). However, the precise passive and active spine mechanisms that promote the linear summation of subthreshold inputs remains unknown. Thus, subthreshold depolarizations in the dendrites that arise from multiple synchronously activated spines—either spatially clustered (Araya et al., 2006a; Gasparini and Magee, 2006; Losonczy and Magee, 2006) or distributed (Gasparini and Magee, 2006; Losonczy and Magee, 2006)—summate linearly before the generation of a dendritic spike (Figures 5, 6).

### Spines as activity-dependent gain modulators: a hypothesis on the true *raison d'etre* for spines

If one of the important functions of spines is to promote the linear integration of subthreshold dendritic depolarizations that are triggered by synchronous, clustered excitatory inputs, then why are excitatory inputs still directed to spines in circumstances when the distance between synapses is large enough to allow linear summation if the inputs were directed directly to the dendritic shaft? One possible reason, and perhaps the true *raison d'etre* for spines, is that they can undergo transient and persistent activity-dependent structural [spine head enlargement (Lang et al., 2004; Matsuzaki et al., 2004; Harvey et al., 2008), spine head reduction (Zhou et al., 2004; Oh et al., 2013; Wiegert and Oertner, 2013), and neck plasticity (Bloodgood and Sabatini, 2005; Grunditz et al., 2008; Tanaka et al., 2008; Araya et al., 2014; Tonnesen et al., 2014)] and molecular changes (Malenka and Bear, 2004; Yasuda, 2006; Harvey et al., 2008; Lee et al., 2009; Murakoshi et al., 2011; Yasuda and Murakoshi, 2011; Lisman et al., 2012) that can modify synaptic strength (Matsuzaki et al., 2004; Araya et al., 2014), and hence alter the gain of pyramidal neuron input/output properties without the need to rewire the network.

Hence, the hypothesis I put forth is that: (1) Sub-threshold depolarizations in the dendrites, arising from multiple synchronously activated spines—either spatially clustered or distributed—summate linearly before the generation of a dendritic spike (Figures 5, 6); and (2) that spine plasticity triggers rapid and reversible changes in synaptic weight and hence in the gain of pyramidal neuron input/output properties, providing an effective and rapid control of the threshold (number of spines activated) required to generate a dendritic or somatic spike (Figure 6).

This more economical way of modifying a neuron's computational power would rely on the ability to modify the passive (e.g., spine morphology) and active (recruitment of voltage-gated channels) properties of spines, influencing the neuron's electrical and biochemical compartmentalization capabilities and providing a fast and effective control of synaptic transmission, storage and integration.

Another prediction for this hypothesis is that the activity-dependent spine changes, although sufficient to change synaptic efficacy, might not be drastic enough to disrupt the linearly integrated sub-threshold dendritic depolarization. This would prevent the sublinear integration of synchronous and clustered inputs that would be observed if inputs impinged on spines with a low *R_N_* or directly onto the dendritic shaft. I based this prediction on the experimental observations showing that linearity of sub-threshold depolarization is protected even when the uncaging of glutamate was directed to clustered spines of different morphologies (Araya et al., 2006a; Gasparini and Magee, 2006; Losonczy and Magee, 2006).

To test this hypothesis, a spatially multiplexed imaging/uncaging tool such as a spatial light modulator (SLM) (Nikolenko et al., 2008) could be employed. This would allow the simultaneous uncaging of glutamate (with single spine resolution) at several spines (up to 30 in a 2P regime) and facilitate study of the role of spines in spatial summation, as well as how plasticity paradigms might affect the input/output gain and/or integration algorithm of sub-threshold depolarizations.

## Spines and disease

In his seminal article “Dendritic spine ‘dysgenesis’ and mental retardation,” Dr. Purpura described the perfect correlation between the degree of mental retardation and the extent of spine morphological aberrations, with dendrites from cortical neurons of retarded children being covered with abnormally long and thin dendritic spines (Purpura, 1974). Since then, many studies have indicated that an important phenotype in many brain disorders is the abnormal shape and density of dendritic spines (see below). As pointed out before, the correlation between form and function of dendritic spines is well accepted; thus the notion that alterations in spine morphology affect synaptic transmission, integration and information storage is not challenged.

Fragile X syndrome (FXS) is the most frequent form of inherited mental retardation (Jacquemont et al., 2007; Hagerman et al., 2010) and the most common known single-gene cause of autism (Wang et al., 2012). FXS is caused by inactivation of the Fragile X Mental retardation 1 (FMR1) gene, which encodes the Fragile X Mental Retardation Protein (FMRP) (Bassell and Warren, 2008; De Rubeis and Bagni, 2010) an RNA-binding protein that has a major role in inhibiting the translation of bound mRNAs, especially at neuronal synapses (Darnell et al., 2011; Wang et al., 2012). At a gross scale the post-mortem brains of FXS patients are almost intact (Reiss et al., 1995; Hallahan et al., 2011). However, at the micro-anatomical level it has been found that FXS is characterized by major alterations to dendritic spines, with abnormally long-necked spines with prominent heads mixed with normal looking spines (Rudelli et al., 1985; Irwin et al., 2001). The assumption was that since these spine aberrations resemble immature spines, the phenotype could be indicative of developmental dendritic deficits. A similar spine phenotype—with long, thin and tortuous spines—was evident in a mouse model of FXS, the Fmr1 KO mice (Irwin et al., 2002; Galvez and Greenough, 2005). In addition, there is evidence in the Fmr1 KO mice that not only is spine morphology impaired, but that spine density is increased (Comery et al., 1997; Galvez and Greenough, 2005; McKinney et al., 2005). Furthermore, it has been demonstrated in these mice that stronger neuronal activity is required to trigger STDP in Fmr1 KO mice (Meredith et al., 2007). The reader is referred to the following reviews on spine density, maturity and plasticity in FXS for further information (Portera-Cailliau, 2012; He and Portera-Cailliau, 2013). Moreover, in Rett syndrome, a disease caused by mutations in the X-linked methyl CpG binding protein 2 (MECP2) and associated with intellectual disabilities (Amir et al., 1999), patients exhibit a drastic reduction in cortical pyramidal neuron spine density (for review see Xu et al., 2014).

A reduction in the number of spines and dendritic impairments has also been observed in aging, psychiatric disorders such as schizophrenia and major depressive disorder, and in neurodegenerative disorders such as Alzheimer's disease (Peters et al., 1998; Fiala et al., 2002; Penzes et al., 2011; Koleske, 2013). The list of diseases in which spine morphology and density have proven important is vast, including not only neurodegenerative or psychiatric disorders, but also diseases like epilepsy in which spine loss is evident (Scheibel et al., 1974; Isokawa, 1997). This is perhaps not surprising since spines are the main gateway of excitatory and some inhibitory information in the brain, and alterations in spine structure and function will likely have huge effects on input transformation in the brain.

In conclusion, the study of spines has proven to be essential for the understanding of synaptic processing, plasticity and integration in pyramidal neurons. In addition, these tiny protrusions are believed to be the pathogenic substrate in neuropsychiatric disorders in which spine structure, density and/or function are impaired. Thus, understanding spine function will not only shed light on how pyramidal neurons and the circuits in which they reside work, but will also provide a new framework for understanding the contribution of spines to various diseases. This may in turn aid the development of novel therapeutic approaches for neurodegenerative disorders such as Alzheimer's disease and Fragile X-syndrome, illnesses in which spine structure and function are impaired.

### Conflict of interest statement

The author declares that the research was conducted in the absence of any commercial or financial relationships that could be construed as a potential conflict of interest.
